# 

**Published:** 1843-12-23

**Authors:** 


### THE MEDICAL EXAMINER,

### And Retrospect of the Medical Sciences.

EDITED BY

MERIDITH CLYMER, M.D

Lecturer on the Institutes of Medicine, Physician to the Philadelphia Hospital, Blockley, Fellow of the College of Physicians, Philadelphia, etc. etc.

VOL. VI.] PHILADELPHIA, DECEMBER 23, 1843. [No. 25.

TO SUBSCRIBERS.

The attention of our subscribers, who are in arrears, is respectfully invited to the terms of subscription. An early attention to our claims is solicited. When it is considered that the Examiner is purely a professional journal, and unsupported by the capital of a publishing house, the propriety of a compliance with our demands will be generally recognized. Funds current in the state where the subscriber resides, will be received.

CONTENTS.

*Clinical Lectures and Reports.—Jefferson Medical College.*

Clinic of Professor Mütter.

Case 1. Tumour of the Nape of the Neck, 289

Case 2. Congenital Icthyosis, - - 289

Case 3. Lupus of the Lower Lid, - - 289

Case 4. Cancer of the Lip, - - - 290

Case 5. Cephalomatous Tumour, - - 290

Clinic of Dr. Pancoast.

Case of Paracentesis Cerebri, - - - 291

*Columbia College, D. C.*

Clinic of Dr. William P. Johnston.

Amaurosis, - - - - - 292

Lenticular Cataract, (Hard,) - - 293

*Philadelphia Hospital.*

THE MEDICAL EXAMINER, AND RETROSPECT OF THE MEDICAL SCIENCES,

IS PUBLISHED EVERY ALTERNATE SATURDAY.

OFFICE AT J. C. TURNPENNY'S, N. E. CORNER OF SPRUCE AND TENTH STREETS.

SUBSCRIPTION PRICE, THREE DOLLARS A YEAR, invariably in advance. Any person enclosing TEN DOLLARS on a solvent bank, *free of postage*, will receive FIVE copies.

Communications and Letters to be addressed (always pre-paid) *in every instance* to the Editor of the Medical Examiner, Philadelphia.

Postmaster's franks can always be obtained for remittances.

This Journal is subject to newspaper postage only.

*Rates of Advertising.*—For first insertion of 20 lines or less, $1—each additional insertion 50 cts. For advertisements occupying a whole page, and inserted yearly or half-yearly, a large reduction from the above rates will be made.

MERRIHEW AND THOMPSON, PRINTERS, 7 CARTER'S ALLEY.

### THE MEDICAL EXAMINER,

AND

### RETROSPECT OF THE MEDICAL SCIENCES.

EDITED BY

MEREDITH CLYMER, M. D.

Lecturer on the Institutes of Medicine, Physician to the Philadelphia Hospital, Fellow of the College of Physicians, &c.

The Seventh Volume of this Journal will commence early in January next.

The success which has attended the present year of the EXAMINER, has induced the Editor to make suitable arrangements by which the value of the Journal will, for the future, be materially increased. A main and distinguishing feature of the work will be, as heretofore, the publication of the Clinical Lectures delivered in this city, in Paris, and other parts of the Union, with Reports from the Hospitals, and Dispensaries of Philadelphia. Original Communications of real value, contributed by many of the most eminent men in the profession, will appear from time to time. The Bibliographical Department will be materially enlarged, and in future Critical and Analytical Reviews of all the prominent Medical Publications in Great Britain, on the continent of Europe, and in this country, will be regularly given. In the Editorial Department all subjects connected with Medical Ethics and Politics will be freely but temperately discussed. It is in the *Retrospect* chiefly, however, that the main improvement and alteration are proposed. A condensed Summary of ALL *the Articles* of a *Practical nature*, from ALL *the Journals, Foreign and Domestic*, prepared with the greatest care, with extracts from books difficult of access, will occupy a large space in each number. The MEDICAL EXAMINER will thus offer the most complete Retrospect of the Medical Sciences now published, and from the short intervals of publication, (*two weeks*,) the profession will be put in immediate possession of every medical fact of importance, on the arrival of the steamer.

The great success attending the publication of the Clinical Lectures of Professor Chomel of Paris, during the present year, has induced the Editor to make arrangements for the publication of a complete course of Lectures on Fractures, by Professor Velpeau. They will be commenced in the first No. of the next volume.

During the present year Clinical Lectures by Professors Chomel, Roux, Rostan, Huguier, of Paris, Horner, Mitchell, Pancoast, Mütter, Drs. Randolph, W. Poyntell Johnston, Clymer of Philadelphia, and Prof. W. P. Johnston, of Washington, D. C., have been published; with Clinical Reports from the Philadelphia, Pennsylvania, and Wills's Hospitals, and the Philadelphia Dispensary. It contains a variety of original papers from many of the most distinguished physicians in the United States; a Parisian Correspondence; a Complete Bibliographical Record of all the new medical publications; numerous Editorials on the principal medical topics of the day, including a series of articles on the condition of the Medical Department of the Navy; together with a complete *Retrospect of the progress of Medicine, Surgery, and the Collateral Sciences*, during the present year.

Since the commencement of the Examiner in 1838, there have been published, besides the usual matter of medical journals, a complete course of Lectures, on Diseases of the Eye, by Professor VELPEAU; a complete course of lectures on the Diagnosis and Treatment of Diseases of the Chest, by Dr. GERHARD; a complete course of lectures on the Exanthematous Diseases, on Gout and Rheumatism, on Diseases of the Larynx and Trachea, by Professor CHAPMAN; a complete course of Clinical Lectures and Hospital Clinics, by Dr. GERHARD; Clinical and other Lectures, on a variety of subjects, by Drs. THOMAS HARRIS, RANDOLPH, GIBSON, HORNER, HARLAN, W. HARRIS, COATES, POYNTELL JOHNSTON, CARSON, CASPER MORRIS, WARRINGTON, of Philadelphia; BRODIE, COOPER, WATSON, SIGMOND, MARSHALL HALL, GRAVES, STOKES, CARSWELL, ALCOCK, PRICHARD, GUTHRIE, TWEEDIE, of the British Schools; VELPEAU, ROUX, CHOMEL, ANDRAL, ORFILA, CAZENAVE, RICORD, GUERIN, GERDY, TROUSSEAU, AMUSSAT, and others of the French and Continental Schools.

The Examiner is published punctually *every alternate Saturday*, at *Three Dollars* a year, invariably in advance. It is printed on thick white paper, with clear new type, double column, imperial octavo.

Any person enclosing TEN DOLLARS (*free of postage*,) on a solvent Bank, will receive FIVE Copies.

Communications and Letters to be addressed *in every instance* (always *pre-paid*) to the Editor of the Medical Examiner, Philadelphia.

☞From the extensive circulation of this Journal it is a desirable medium for general advertisers. Advertisements inserted on the cover on the most reasonable terms.

Postmasters' franks can always be obtained for remittances.

This Journal is subject to newspaper postage only.

The five back volumes may be had, bound, at reduced prices.

Subscriptions received at the office, J. C. TURNPENNY'S, N.E. corner of Spruce and Tenth Streets, Philad.

